# Fear of Falling Score Is a Predictor of Falls in Community-Dwelling Pre-Frail and Frail Older People

**DOI:** 10.3390/healthcare11152132

**Published:** 2023-07-26

**Authors:** Lucía Prieto-Contreras, Francisco M. Martínez-Arnau, David Sancho-Cantus, Laura Cubero-Plazas, Pilar Pérez-Ros

**Affiliations:** 1 Nursing Department, Universidad Católica de Valencia San Vicente Mártir, Espartero 7, 46007 Valencia, Spain; lucia.prieto@ucv.es (L.P.-C.); david.sancho@ucv.es (D.S.-C.); laura.cubero@ucv.es (L.C.-P.); 2 Department of Physiotherapy, University of Valencia, Gascó Oliag 5, 46010 Valencia, Spain; 3 Frailty and Cognitive Impairment Organized Group (FROG), University of Valencia, 46010 Valencia, Spain; maria.p.perez-ros@uv.es; 4 Department of Nursing, University of Valencia, Menéndez Pelayo s/n, 46010 Valencia, Spain

**Keywords:** frailty, falls, community-dwelling, fear of falling, aged

## Abstract

Identifying frail older people at risk of falling is a priority in order to apply preventive strategies. This cross-sectional study included community-dwelling pre-frail and frail people (Fried’s criteria) aged 70 years and older to assess the prevalence of falls and identify screening strategies based on comprehensive geriatric assessments to detect an increased risk of falling and recurrent falling in community-dwelling frail and pre-frail old people. Of the 229 participants, 121 (54.9%) had fallen in the previous 12 months, and 20 of these (16.5%) were recurrent fallers (≥2 falls). A score of 20 points or more on the Falls Efficacy Scale International was predictive of falling (area under the receiver-operating characteristics curve 0.67, 95% confidence interval: 0.61–0.74, *p* < 0.001) with a sensitivity of 51.7% and a specificity of 73.9%. Polypharmacy, Short Physical Performance Battery score of 8 points or less, and Falls Efficacy Scale International score of 20 points or more show an area under the curve of 0.78 (95% confidence interval: 0.67–0.89, *p* < 0.001) for recurrent falling.

## 1. Introduction

The growing population of older people worldwide, along with the numerous health problems associated with advanced age [1], has increased the urgency of establishing preventive strategies to minimize the prevalence of social and health impacts. Frailty is a syndrome characterized by increased vulnerability, stemming from a decline in the reserve and function of several physiological systems [2,3]; its estimated prevalence ranges anywhere from 5.8% to 35% in different studies [4,5]. As a topic of research and target of clinical practice, frailty has seen a dramatic surge in interest in recent years, and although there is currently no consensus criteria for defining it [6,7,8], Fried’s physical criteria, including grip strength and gait speed, among others [9], are usually used in older people.

One of the main geriatric syndromes related to frailty is falling, the etiology of which is multifactorial [10,11]. Chronic diseases such as cancer, diabetes, or depression, along with sociodemographic, physical, biological, and lifestyle-related factors, significantly increase the risk of falling in community-dwelling older people [6,7,8], representing the second cause of death in this population, together with accidental injuries [12].

A recent systematic review of prospective studies revealed that the risk of falls, bone fractures, disability, dementia, hospitalization, and death were much higher among frail people [13]. This population has a higher prevalence of falls than pre-frail and robust older people, but they are also the ones who benefit most from fall prevention programs, so identifying them and implementing preventive strategies as early as possible is crucial for health promotion [14].

The comprehensive geriatric assessment (CGA) is a key tool for preventing falls [4], since its performance contributes to reducing admissions to nursing homes, the risk of falls, pressure ulcers, delirium, and risk of frailty in community-dwelling older people [15]. Often performed by nurses, the CGA consists of an evaluation of the physical, functional, psychological, and social domains of the older person [16]. The physical or clinical domain covers the presence of comorbidity, daily drug use, the perception of pain, analytical and anthropometric data; the functional domain covers the ability to perform basic, instrumental, and advanced activities of daily living together with gait and balance; the psychological domain covers cognition or the possible presence of fears, anxiety, or depression; and the social domain covers a person’s resources, social support, and feelings of loneliness. Knowing how each of these spheres is affected allows us to identify geriatric syndromes early on and to act on them. The CGA is described as the best method for assessing the health state and treatment needs of older persons in a new chapter of Harrison’s *Principles of Internal Medicine* [17].

In this context, assessing the risk of falls is complex. Considerations must include both intrinsic risk factors related to the person, such as age, female sex, comorbidity, visual or hearing deficits, gait and balance disorders, loss of functionality, polypharmacy or use of drugs that increase the risk of falls (e.g., hypotensives or central nervous system depressants), and fear of falling (FoF), as well as extrinsic risks, including environmental factors such as architectural barriers, lighting in the home or on the streets, and footwear [18].

A well-designed CGA will obtain all this information. The use of validated assessment scales is very common in the CGA, since it allows the use of standardized instruments to obtain valid information to identify a syndrome or problem and, thanks to the score obtained, to be able to screen quickly. In the case of a CGA, there are assessment instruments for most geriatric syndromes. The final design of the CGA will depend on the time available, the type of environment in which the assessment is carried out (community, hospital, long-term care), the presence of cognitive impairment, and the help of relatives or caregivers who can provide information, among other aspects [19].

Because the main barrier to implementing a CGA is the time needed to perform it, abbreviated CGAs have been designed in different settings [19]. Moreover, some assessment instruments can be used to identify more than one impairment, depending on the cutoff point or the specific population. For example, the score obtained in the Time up and Go helps to identify the risk of falls and is also useful as a frailty criterion. Therefore, assessment instruments that provide valid information for several geriatric syndromes, including, for instance, the risk of falls, reduces the time required for the CGA [20]. Moreover, frail older people in community settings benefit the most from a CGA [21]. Assessments in settings such as hospital emergency departments or intensive care units are more challenging [22].

This study aims to assess the prevalence of falling and identify screening strategies based on comprehensive geriatric assessments to detect an increased risk of falling and recurrent falling in community-dwelling frail and pre-frail old people.

## 2. Materials and Methods

### 2.1. Study Design and Participants

This cross-sectional study took place in primary care centers and social centers for the elderly in the health department of La Ribera (Valencia, Spain). Recruitment and assessment were performed from August to December 2016. The catchment population was 20,613 during the study period, of which 15% were aged 70 or older. Considering a prevalence of 30% for falls and of 10.7% for frailty in older people [23], for an error of 3% (α = 5), a sample size of 226 participants was calculated. We initially recruited 432 community-dwelling older people, of whom 115 did not meet frailty criteria, 56 refused to participate, and 32 did not sign the informed consent. The final sample size was 229 participants.

The research team consisted of three nurses and one physiotherapist, all experienced in studies in older people, who carried out the CGAs. We first contacted the directors of eligible centers, then held meetings with the nursing staff to explain the inclusion criteria and resolve any concerns they had, as staff in regular contact with the study population. Following recruitment, each participant was informed of the study aims and signed informed consent. They were then assigned an identification number for the study, consisting of the first three letters of their municipality and a number.

Inclusion criteria were people aged 70 years or older who met criteria for frailty or pre-frailty, were able to walk independently (with or without walking aid), and regularly received care in the primary care centers of La Ribera health department. Exclusion criteria were life expectancy of less than 6 months, hearing loss that prevented the assessment, or diagnosis of severe psychiatric disorders or moderate to severe cognitive impairment.

Frailty was defined using Fried’s criteria [24]. Participants were classified as frail if they met three out of the following five criteria: unintentional weight loss, exhaustion, weakness, slow walking speed, and limited physical activity. Prefrailty was described as meeting one or both of the aforementioned criteria [25]. In terms of how these criteria were defined, unintentional weight loss was defined as losing 4.5 kg, or 5% of body weight, in the last year (as assessed by direct weight measurement). Exhaustion was defined according to self-report: “I felt that everything I did was an effort” or “I could not get going.” Standardized protocols were used to measure the dominant hand’s strength using a 100 kg dynamometer (Smedley S, TTM, Tokyo, Japan) while the patient was seated. Weakness was determined by using grip strength cutoffs in the lowest 20% stratified for gender and body mass index (BMI). Gait speed was assessed in a 4.5 m corridor as a dichotomous variable, with low speed defined as < 0.8 m/s. Finally, individuals were classified as having low physical activity according to the weighted amount of kilocalories expended per week (men, 383 kcal/week and women, 270 kcal/week. The assessment of physical activity was based on modified Minnesota Leisure Time Activities Questionnaire, asking about walking (w = 3.5), strenuous household chores (w = 4.5), strenuous outdoor chores (w = 4.5), dancing (w = 5.5), bowling (w = 3.0), and exercise (w = 4.5). To compute kcals expended per week, we used the following formula: Kcals (Kilocalories/week) = w × Frequency (sessions per week) × Duration per session (minutes) × Body Weight (kg)/60, where w is the task-specific MET intensity score.

The research ethics committee of the Hospital Universitario La Ribera approved the study (date October 2015), which complied with the principles set out in the Declaration of Helsinki. All the participants signed informed consent before inclusion in the study and the statistical processing of the data.

### 2.2. Data Collection

All participants underwent a CGA. Sociodemographic variables (age, sex, cohabitation) were included. Age was considered a continuous variable, but an age category for those over 80 years old was created, as this group was considered at potentially higher risk of falls.

### 2.3. Clinical Assessment

Patients’ electronic medical records were reviewed to collect data on comorbidities: osteoporosis, hypertension, hyperlipidemia, diabetes mellitus, heart failure, mixed anxiety–depressive disorder, and orthostatic hypotension. We also recorded the main classes of prescription drugs related to the risk of falls, such as antiplatelets, beta-blockers, alpha-blockers, angiotensin-converting enzyme (ACE) inhibitors/angiotensin II receptor antagonists (ARAII), diuretics, benzodiazepines, and antidepressants. The number of drugs prescribed per day was also analyzed, with polypharmacy defined as the prescription of 5 or more drugs per day [26].

### 2.4. Functional Assessment

The functional assessment included the following instruments:

Barthel Index (BI) [27]. The BI is an ordinal scale assessing independence in performing 10 basic activities of daily living. It is scored from 0 points (total dependency in activities of daily living) to 100 points (total independence).

Lawton Instrumental Activities of Daily Living (IADL) [28,29]. The Lawton IADL scale measures eight functional areas. Patients are scored according to their highest level of functioning in that category. The summary score ranges from 0 to 8, with a higher score being synonymous with greater independence.

Short Physical Performance Battery (SPPB) [30]. This instrument consists of three tests: balance, gait speed, and repeated chair stands (×5). Each of the three tests is scored from 0 (worst) to 4 (best), for a total score ranging from 0 to 12. A score of 8 or less is associated with frailty and falls [31].

### 2.5. Emotional Assessment

The Yesavage Geriatric Depression Scale (GDS) [32] was used for the emotional assessment. It consists of 15 items. Depending on age, education, and complaints, a score of 0 to 4 points is regarded as normal, while a score of 5 or more indicates depressive symptoms.

### 2.6. Fall Risk Assessment

A fall was considered as an incident that causes a person to unintentionally rest on the ground, a floor, or another lower level. Participants verbally indicated how many falls they had experienced in the previous 12 months, and health professionals retrieved any additional data that was recorded from visits to the hospital’s emergency department following a fall.

The prevalence of falls was calculated as the number of falls in the sample in the previous 12 months out of the total sample. The number of fallers was the number of people having at least one fall in the previous 12 months out of the total sample. The number of recurrent fallers was the number of people suffering two or more falls in the previous 12 months out of the total sample.

The number of risk factors for falls [33] was collected, as was the fear of falls, using the Falls Efficacy Scale International (FES-I) [34]. The 16-item questionnaire assesses self-efficacy and confidence in carrying out activities of daily living, on a scale of 16 (no concern about falling) to 64 (severe concern about falling).

### 2.7. Statistical Analysis

All data entered into the database were verified by an independent second person. Descriptive statistics were expressed as mean and standard deviation (SD) for normally distributed continuous variables and relative frequencies for categorical variables. Two-tailed *p*-values of less than 0.05 were considered statistically significant.

The odds ratio (OR) was used to depict the relationship between falls and the various risk factors taken into consideration. A receiver operating characteristics (ROC) curve was used to determine the area under the curve (AUC) for the recurrent fallers model along with the cutoff value, sensitivity, and specificity of the FES-I.

To investigate the significance of the risk factors, defined in accordance with the falls observed during the preceding 12 months, two binary logistic regression models, adjusted for age and gender, were fitted for fallers and recurrent fallers. The first complete model, with all the variables in the bivariable analysis, was significantly associated with the presence of falls over the previous year. In a subsequent phase, all the variables that failed to create a substantial change when excluded (defined as the lack of an adjusted effect of >10%), as well as those that did not improve the standard error, were removed from the model. When two or more subsets of variables with the same goodness of fit were found, the authors aimed to reach a consensus. Age, gender, cohabitation, polypharmacy, number of fall risk factors, SPPB ≤ 8 points, and FES-I ≥ 20 points were the variables that were included in the backwards Wald model.

The variable in the second model compared recurrent fallers (2 or more falls) with non-fallers and isolated fallers (1 fall) from the preceding 12-month period, as opposed to the first model’s dichotomous variable (1 or 0), which compared fallers with non-fallers. Age, gender, polypharmacy, SPPB ≤ 8 points, and FES-I ≥ 20 points were the variables included in the backwards Wald model.

Analyses were undertaken with SPSS version 26.0 for Windows (IBM Corp., Armonk, NY, USA) and Jamovi 2.2.5 statistical packages.

## 3. Results

The final sample consisted of 229 participants; 41% (n = 94) were pre-frail and 59% (n = 135) frail.

The prevalence of falls in the previous 12 months was 54.9% (n = 121); 38.8% (n = 89) of participants were fallers (≥1 fall), and 16.5% of these (n = 20) were recurrent fallers (≥2 falls), while the remaining 61.2% (n = 140) were non-fallers. The mean number of falls over the previous year in fallers was 1.31 (SD 0.86).

There was a predominance of women in the sample, and the mean age was over 77 years in both fallers and non-fallers. Women were also at higher risk of falls. No differences were found according to cohabitation, comorbidity, polypharmacy, or drug classes (Table 1).

The frail older people included in the sample showed high functionality for basic and instrumental activities of daily living, with no differences between groups. No differences were found in SPPB scores. The mean score on the Yesavage scale was below the cutoff for depressive symptoms. Significant differences were found in the FES-I scale for FoF (Table 1).

**Figure 1:**
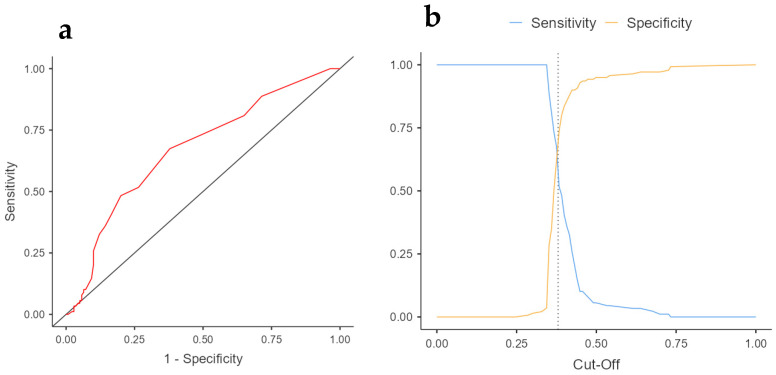
FES-I area under the curve and cutoff for community-dwelling frail old people. (**a**) FES-I area under the curve for frail old people; (**b**) FES-I cutoff for frail old people. The dashed line represents the highest point of sensitivity and specificity, indicating the cut-off value.

After analyzing the participant characteristics and finding differences in the FES-I score, a score analysis was performed to identify the best cutoff for detecting falls in frail and pre-frail older people. A score of 20 points or more showed the best performance, with an AUC of 0.67 (95% CI 0.61–0.74, *p* < 0.001), sensitivity of 51.7%, and specificity of 73.9% (Figure 1). Those with 20 points or more showed an increased risk of falls (OR 3.39; 95% CI 1.94, 5.94; *p* < 0.001). There was a higher percentage of women with FES-I ≥ 20 points than men (85.8 [n = 97] vs. 14.2 [n = 16]. In addition, a higher risk of FoF was observed in women compared to men, OR 1.56; 95% CI 1.30–1.86, *p* < 0.001.

**Figure 2:**
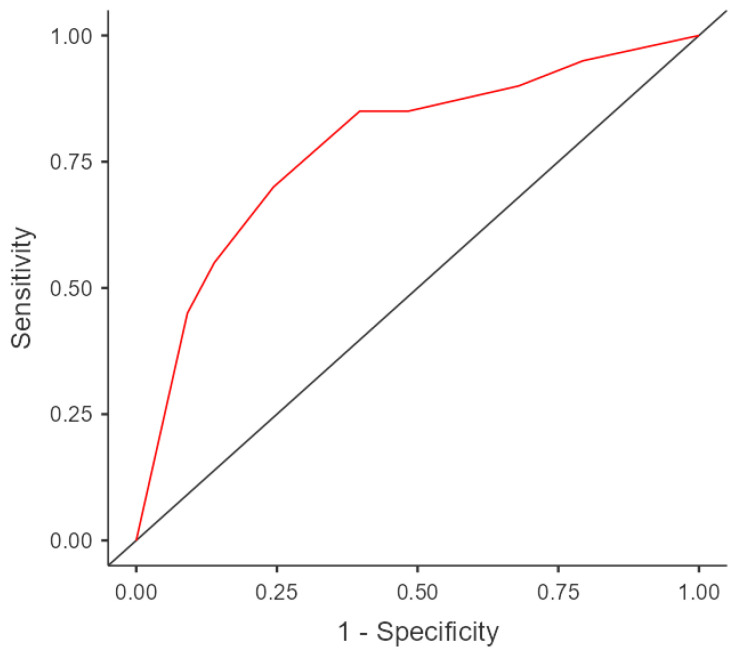
ROC curve for recurrent fallers.

After performing a binary logistic regression for the group of recurrent fallers, the following variables were included in the model: polypharmacy (≥5 daily active ingredients), SPPB score of 8 points or less, and FES-I score of 20 points or more. The model classified 91.3% of the cases well, with an R^2^ = 0.197 (*p* < 0.001; Table 2). For the variables included in the model, the AUC was 0.78 (95% CI 0.67–0.89, *p* < 0.001; Figure 2).

## 4. Discussion

Frail people are at higher risk of falls due to the multisystem deterioration derived from the frailty process. The coexistence of both geriatric syndromes, frailty and falling, increases the risk of dependency and loss of quality of life [35]. Detecting and preventing these geriatric syndromes to maintain functionality during the aging process is a real challenge [36]. This study assessed the prevalence of falls, their risk factors, and the possible existence of CGA instruments that aid in the detection of falls in community-dwelling frail and pre-frail old people. Altogether, the prevalence of falls was 54.9%. Being a woman and having a score of 20 points or more on the FES-I fear of falls scale were predictors of falls in this population. For recurrent fallers (≥2 falls in the previous 12 months), predictors were polypharmacy (≥5 or more daily drugs), an SPPB score of 8 points or less, and an FES-I score of 20 or more.

Our cohort has similar sociodemographic characteristics to other studies in community-dwelling older people, with a predominance of women, who present the highest risk of falls [37]. No difference in fall risk was observed in relation to age or cohabitation, probably due to the high functionality of the study participants [38]. Likewise, the prevalence of comorbidities was similar to other community samples, with a greater presence of hypertension and osteoporosis [39]. Despite evidence that certain diseases increase the risk of falls, we did not observe any association in our results. Again, this is likely because of our cohort’s high functional state and/or the good control of their chronic conditions, with no significant relationship between fallers and comorbidities.

An increasing number of drug prescriptions are observed in parallel with the aging of the population [40]. Polypharmacy is a risk factor for frailty and falls, so it is not surprising that in frail older people the increased risk is so high [41].

Although polypharmacy is not synonymous with inappropriate prescription, the two concepts are strongly associated. However, the incorporation of incentives for prescriptions and a greater awareness about the potential harms of polypharmacy among prescribers could improve this situation [42].

Our sample showed high functionality for independence in basic and instrumental activities of daily living. However, physical performance outcomes according to the SPPB scale were not as positive, with a mean score of 8 out of 12 points. We did not observe differences between fallers and non-fallers in the SPPB score, although the literature indicates that the SPPB score is related to falls in older people [30]. This aspect may be influenced by functional independence and other factors such as level of education, female sex, or quality of life, which could minimize this risk [43].

Scientific evidence highlights the need to screen for frailty [44], especially in the context of those with a higher risk of falling. However, comprehensive assessments are not feasible in daily clinical practice due to lack of time or limited knowledge of the instruments [45]. A well-designed CGA with adequate instruments can generate health benefits in older people, helping to reduce falls and frailty, improving mental health outcomes, and increasing quality of life [46]. Knowing whether existing tools included in the CGA could serve to screen frail people for risk of falls would enable more complete subsequent assessments in vulnerable people [47].

In community-dwelling older adults, prevalence rates for FoF vary from 21.0% to 85.0% among fallers and 33.0% to 46.0% among those with no history of falls. FoF can be viewed as a protective reaction because it encourages an individual to be more aware of their surroundings. On the other hand, it can affect a person’s physical and psychosocial well-being when it is unreasonable and overstated because it causes them to restrict or avoid activities. FoF has been associated with a number of adverse outcomes, including a decrease in functionality and social interactions, increased risk of depression, poor health-related quality of life, and all-cause death [48].

Our results indicate that FoF is associated with a higher risk of falling in community-dwelling frail and pre-frail older people. A score of 20 points or more on the FES-I scale is indicative of a three-fold risk of falling in this population, signaling the need for specific prevention strategies [49].

A recent systematic review of longitudinal and cross-sectional studies concluded that FoF and frailty are related in older persons who live in their communities. The understanding of this link is crucial for clinical practice because it helps health professionals create early frailty screening methods and strategies for treatment, prevention, and health promotion. Additionally, it can support health education and prevention initiatives targeted at lowering FoF and, as a result, the onset of frailty and its detrimental effects [37].

The literature differentiates between risk factors for falls and for recurrent falls; we analyzed the possible factors associated with recurrent falls [37,49,50]. In our cohort, polypharmacy, SPPB score of 8 points or less, and FES-I of 20 points or more indicate increased risk. Other authors have reported that people with an SPPB score under 8 points have a higher risk of developing FoF in the following two years [43]. This could explain the relationship between the SPPB and the FES-I in recurrent fallers. The SPPB is a quick and easy-to-use tool in the primary care setting [51], so it could be used together with the self-reported FES-I scale to identify frail people at risk of falls in this setting. The risk for a new fall in recurrent fallers is therefore increased [52], and the risks detected are similar to those found in the frail older population [18]. The presence of polypharmacy makes sense in frail patients with recurrent falls, who also show worse physical performance, as assessed with SPPB. A CGA would be the most appropriate screening tool in this situation, given the concurrence of physical maladies (as indicated by polypharmacy), functional performance (SPPB), and psychological well-being (fear of falls, assessed with FES-I).

Despite the evidence on the positive health outcomes resulting from CGAs in frail, community-dwelling [53], and hospital populations [54], there is a great need to carry out longitudinal studies on the effects of performing a CGA in the general population of community-dwelling older people [55]. However, the heterogeneity of the included assessment tools makes it difficult to compare and analyze the effectiveness of the interventions carried out in the community, regardless of the population to which they are applied [55].

### Limitations

The study’s cross-sectional design precludes any causal analysis of risk factors. It was not possible to analyze the influence of some of individual risk factors due to the lack of disaggregated data. Moreover, the descriptions of falling were based on self-report, so it was not possible to contrast these with an objective observation in order to determine the cause of the fall.

## 5. Conclusions

The prevalence of falls in community-dwelling frail and pre-frail old people was 54.9%. A cutoff of 20 points on the FES-I scale, assessing fear of falling, was predictive of increased risk of falling in this population. In addition, polypharmacy, an SPPB score of 8 points or less, and an FES-I score of 20 points or more were predictive of recurrent falling. Screening frail and pre-frail people for fall risk is possible using the assessment instruments included in the CGA.

## Figures and Tables

**Table 1:** Characteristics of the sample according to the falls group.

	Fallers (n = 89) n (%)	Non-Fallers (n = 140) n (%)	OR (95% CI) *	*p*-Value
Age, years, mean (SD)	77.54 (4.53)	77.88 (5.07)	MD * 0.34 (−0.96, 1.64)	0.61
Age ≥ 80 years	29 (32.6)	49 (35.0)	1.11 (0.63, 1.95)	0.71
Sex				
Male	20 (22.5)	48 (34.3)	—	—
Female	69 (77.5)	92 (65.7)	1.8 (0.98, 3.3)	0.049
Cohabitation				
With partner, adult child, or caregiver	59 (66.3)	98 (70.0)	—	—
Alone	30 (33.7)	42 (30.0)	1.19 (0.67, 2.09)	0.56
Comorbidities				
Hypertension	63 (70.8)	98 (70.0)	1.03 (0.58, 1.86)	0.90
Diabetes mellitus	23 (25.8)	42 (30.0)	0.80 (0.44, 1.47)	0.49
Dyslipidemia	38 (42.7)	59 (42.1)	1.01 (0.59, 1.74)	0.95
Mixed anxiety–depressive disorder	42 (47.2)	57 (40.7)	1.27 (0.74, 2.17)	0.38
Orthostatic hypotension	8 (9.0)	21 (15.0)	0.56 (0.23, 1.32)	0.17
Hearth failure	13.5 (12)	13 (9.2)	1.53 (0.66, 3.54)	0.32
Osteoporosis	28.1 (25)	41 (29.3)	0.93 (0.52, 1.69)	0.83
Drug treatments				
Polypharmacy	47 (52.8)	70 (50.0)	1.12 (0.66, 1.9)	0.68
Antiplatelets	26 (29.2)	46 (32.9)	0.84 (0.47, 0.15)	0.56
Beta-blockers	16 (18.0)	20 (14.3)	1.31 (0.64, 2.69)	0.46
Alpha-blockers	9 (10.1)	12 (8.6)	1.20 (0.48, 2.97)	0.70
ACE inhibitors/ARAII	52 (58.4)	86 (61.4)	0.91 (0.53, 1.56)	0.75
Diuretics	27 (30.3)	51 (36.4)	0.76 (0.43, 1.34)	0.34
Benzodiazepines	23 (25.8)	47 (33.6)	0.69 (0.38, 1.24)	0.21
Antidepressants	19 (21.4)	19 (13.6)	1.72 (0.86, 3.48)	0.13
**Comprehensive Geriatric Assessment**	**Fallers (n = 89)** **Mean (SD)**	**Non-Fallers (n = 140)** **Mean (SD)**	MD (95% CI) *	***p*-Value**
Barthel Index, points	91.29 (13.64)	92.96 (11.04)	−1.67 (−4.90, 1.56)	0.31
Lawton Index, points	7.15 (1.55)	6.91 (1.73)	0.23 (−0.21, 0.67)	0.31
SPPB, points	8.37 (2.97)	8.95 (3.07)	−0.45 (−1.28, 0.38)	0.29
SPPB ≤ 8 points, n (%)	40 (44.9)	43 (30.7)	OR * 1.84 (1.06–3.19)	**0.029**
Yesavage, points	3.17 (3.08)	2.61 (2.82)	0.55 (−0.23, 1.34)	0.16
FES-I, points	23.16 (8.1)	20.59 (8.9)	2.56 (0.25, 4.88)	**0.030**
FES-I ≥ 20 points, n (%)	60 (67.4)	53 (37.9)	OR * 3.39 (1.94–5.94)	**<0.001**
Risk factors for falls, number	5.61 (2.64)	4.56 (3.09)	1.04 (0.20, 1.74)	0.13

* Unless otherwise noted, ACE: angiotensin-converting-enzyme; ARAII: angiotensin II receptor antagonists; CI: confidence interval; FES-I: Falls Efficacy Scale International; MD: mean difference; SD: standard deviation; SPPB: Short Physical Performance Battery; Bold: statistically significant items.

**Table 2:** Predictive model for frail, recurrent fallers.

Variable *	OR (95%CI)	*p*-Value
Polypharmacy	4.21 (1.49–11.9)	0.007
SPPB ≤ 8 points	3.30 (1.11–9.79)	0.032
FES-I ≥ 20 points	3.25 (1.01–10.44)	0.048

CI: Confidence Interval; FES-I: Falls Efficacy Scale International; SPPB: Short Physical Performance Battery; * Backwards Wald model, adjusted for age and gender.

## Data Availability

The data presented in this study are available on request from the corresponding author. The data are not publicly available due to privacy.

## References

[1] Ofori-Asenso R., Chin K.L., Mazidi M., Zomer E., Ilomaki J., Zullo A.R., Gasevic D., Ademi Z., Korhonen M.J., LoGiudice D. (2019). Global Incidence of Frailty and Prefrailty Among Community-Dwelling Older Adults: A Systematic Review and Meta-Analysis. JAMA Netw. Open.

[2] Cappleman A.S., Thiamwong L. (2020). Fear of Falling Assessment and Interventions in Community-Dwelling Older Adults: A Mixed Methods Case-Series. Clin. Gerontol..

[3] Thomas E., Battaglia G., Patti A., Brusa J., Leonardi V., Palma A., Bellafiore M. (2019). Physical Activity Programs for Balance and Fall Prevention in Elderly: A Systematic Review. Medicine.

[4] Cheng M.-H., Chang S.-F. (2017). Frailty as a Risk Factor for Falls Among Community Dwelling People: Evidence From a Meta-Analysis: Falls With Frailty. J. Nurs. Scholarsh..

[5] Tan V., Chen C., Merchant R.A. (2022). Association of Social Determinants of Health with Frailty, Cognitive Impairment, and Self-Rated Health among Older Adults. PLoS ONE.

[6] Jenkins N.D., Welstead M., Stirland L., Hoogendijk E.O., Armstrong J.J., Robitaille A., Muniz-Terrera G. (2023). Frailty Trajectories and Associated Factors in the Years Prior to Death: Evidence from 14 Countries in the Survey of Health, Aging and Retirement in Europe. BMC Geriatr..

[7] Shibata A., Suzuki A., Takahashi K. (2023). Gender Differences in Socio-Demographic Factors Associated with Pre-Frailty in Japanese Rural Community-Dwelling Older Adults: A Cross-Sectional Study. Int. J. Environ. Res. Public Health.

[8] While A. (2023). Minimising Frailty and Its Consequences. Br. J. Community Nurs..

[9] Fried L.P., Tangen C.M., Walston J., Newman A.B., Hirsch C., Gottdiener J., Seeman T., Tracy R., Kop W.J., Burke G. (2001). Frailty in Older Adults: Evidence for a Phenotype. J. Gerontol. A Biol. Sci. Med. Sci..

[10] Davodi S.R., Zendehtalab H., Zare M., Behnam Vashani H. (2023). Effect of Health Promotion Interventions in Active Aging in the Elderly: A Randomized Controlled Trial. Int. J. Community Based Nurs. Midwifery.

[11] Liu T., Liu H., You S. (2023). Analysis of the Impact of Environmental Perception on the Health Status of Middle-Aged and Older Adults: A Study Based on CFPS 2020 Data. Int. J. Environ. Res. Public Health.

[12] Salm C., Sauer J., Binder N., Pfefferle A., Sofroniou M., Metzner G., Farin-Glattacker E., Voigt-Radloff S., Maun A. (2022). Over- and under-Prescribing, and Their Association with Functional Disability in Older Patients at Risk of Further Decline in Germany—A Cross-Sectional Survey Conducted as Part of a Randomised Comparative Effectiveness Trial. BMC Geriatr..

[13] Chu W., Chang S.-F., Ho H.-Y. (2021). Adverse Health Effects of Frailty: Systematic Review and Meta-Analysis of Middle-Aged and Older Adults with Implications for Evidence-Based Practice. Worldviews Evid. Based Nurs..

[14] Pérez-Ros P., Vila-Candel R., Martínez-Arnau F.M. (2020). A Home-Based Exercise Program Focused on Proprioception to Reduce Falls in Frail and Pre-Frail Community-Dwelling Older Adults. Geriatr. Nurs..

[15] Veronese N., Custodero C., Demurtas J., Smith L., Barbagallo M., Maggi S., Cella A., Vanacore N., Aprile P.L., Ferrucci L. (2022). Comprehensive Geriatric Assessment in Older People: An Umbrella Review of Health Outcomes. Age Ageing.

[16] Rubenstein L.Z., Stuck A.E., Siu A.L., Wieland D. (1991). Impacts of Geriatric Evaluation and Management Programs on Defined Outcomes: Overview of the Evidence. J. Am. Geriatr. Soc..

[17] Ouslander J.G., Reyes B., Jameson J.L., Fauci A.S., Kasper D.L., Hauser S.L., Longo D.L., Loscalzo J. (2018). Clinical Problems Associated with the Aging Process. Harrison’s Principles of Internal Medicine.

[18] Jehu D.A., Davis J.C., Falck R.S., Bennett K.J., Tai D., Souza M.F., Cavalcante B.R., Zhao M., Liu-Ambrose T. (2021). Risk Factors for Recurrent Falls in Older Adults: A Systematic Review with Meta-Analysis. Maturitas.

[19] Park S.-H. (2018). Tools for Assessing Fall Risk in the Elderly: A Systematic Review and Meta-Analysis. Aging Clin. Exp. Res..

[20] Lee H., Lee E., Jang I.Y. (2020). Frailty and Comprehensive Geriatric Assessment. J. Korean Med. Sci..

[21] Turner G., Clegg A., British Geriatrics Society, Age UK, Royal College of General Practioners (2014). Best Practice Guidelines for the Management of Frailty: A British Geriatrics Society, Age UK and Royal College of General Practitioners Report. Age Ageing.

[22] Malik S., Parikh H., Shah N., Anand S., Gupta S. (2019). Non-Invasive Platform to Estimate Fasting Blood Glucose Levels from Salivary Electrochemical Parameters. Healthc. Technol. Lett..

[23] Kojima G., Iliffe S., Jivraj S., Walters K. (2016). Association between Frailty and Quality of Life among Community-Dwelling Older People: A Systematic Review and Meta-Analysis. J. Epidemiol. Community Health.

[24] Fried L.P., Ferrucci L., Darer J., Williamson J.D., Anderson G. (2004). Untangling the Concepts of Disability, Frailty, and Comorbidity: Implications for Improved Targeting and Care. J. Gerontol. A Biol. Sci. Med. Sci..

[25] Xue Q.-L. (2011). The Frailty Syndrome: Definition and Natural History. Clin. Geriatr. Med..

[26] Xu Q., Ou X., Li J. (2022). The Risk of Falls among the Aging Population: A Systematic Review and Meta-Analysis. Front. Public Health.

[27] Mahoney F.I., Barthel D.W. (1965). Functional evaluation: The Barthel Index. Md. State Med. J..

[28] Lawton M.P. (1988). Scales to Measure Competence in Everyday Activities. Psychopharmacol. Bull..

[29] Vergara I., Bilbao A., Orive M., Garcia-Gutierrez S., Navarro G., Quintana J. (2012). Validation of the Spanish Version of the Lawton IADL Scale for Its Application in Elderly People. Health Qual. Life Outcomes.

[30] Lauretani F., Ticinesi A., Gionti L., Prati B., Nouvenne A., Tana C., Meschi T., Maggio M. (2019). Short-Physical Performance Battery (SPPB) Score Is Associated with Falls in Older Outpatients. Aging Clin. Exp. Res..

[31] Acosta-Benito M.Á., Martín-Lesende I. (2022). Fragilidad en atención primaria: Diagnóstico y manejo multidisciplinar. Atención Primaria.

[32] Yesavage J.A., Brink T.L., Rose T.L., Lum O., Huang V., Adey M., Leirer V.O. (1982). Development and Validation of a Geriatric Depression Screening Scale: A Preliminary Report. J. Psychiatr. Res..

[33] Ambrose A.F., Paul G., Hausdorff J.M. (2013). Risk Factors for Falls among Older Adults: A Review of the Literature. Maturitas.

[34] Yardley L., Beyer N., Hauer K., Kempen G., Piot-Ziegler C., Todd C. (2005). Development and Initial Validation of the Falls Efficacy Scale-International (FES-I). Age Ageing.

[35] Fhon J.R.S., Rodrigues R.A.P., Neira W.F., Huayta V.M.R., Robazzi M.L.d.C.C., Universidade de São Paulo, Universidad de Ciencias y Humanidades, Universidad Nacional Mayor de San Marcos (2016). Fall and Its Association with the Frailty Syndrome in the Elderly: Systematic Review with Meta-Analysis. Rev. Esc. Enferm. USP.

[36] Wleklik M., Uchmanowicz I., Jankowska E.A., Vitale C., Lisiak M., Drozd M., Pobrotyn P., Tkaczyszyn M., Lee C. (2020). Multidimensional Approach to Frailty. Front. Psychol..

[37] de Souza L.F., Canever J.B., de Moreira B.S., Danielewicz A.L., de Avelar N.C.P. (2022). Association Between Fear of Falling and Frailty in Community-Dwelling Older Adults: A Systematic Review. Clin. Interv. Aging.

[38] Lage I., Braga F., Almendra M., Meneses F., Teixeira L., Araujo O. (2022). Falls in Older Persons Living Alone: The Role of Individual, Social and Environmental Factors. Enfermería Clínica.

[39] Hanlon P., Nicholl B.I., Jani B.D., Lee D., McQueenie R., Mair F.S. (2018). Frailty and Pre-Frailty in Middle-Aged and Older Adults and Its Association with Multimorbidity and Mortality: A Prospective Analysis of 493 737 UK Biobank Participants. Lancet Public Health.

[40] Suzuki Y., Shiraishi N., Komiya H., Sakakibara M., Akishita M., Kuzuya M. (2022). Potentially Inappropriate Medications Increase While Prevalence of Polypharmacy/Hyperpolypharmacy Decreases in Japan: A Comparison of Nationwide Prescribing Data. Arch. Gerontol. Geriatr..

[41] Wang X., Hu J., Wu D. (2022). Risk Factors for Frailty in Older Adults. Medicine.

[42] Esteban Jiménez Ó., Arroyo Aniés M.P., Vicens Caldentey C., González Rubio F., Hernández Rodríguez M.Á., Sempere Manuel M. (2018). Deprescribiendo para mejorar la salud de las personas o cuando deprescribir puede ser la mejor medicina. Atención Primaria.

[43] Curcio C.-L., Wu Y.Y., Vafaei A., de Barbosa J.F.S., Guerra R., Guralnik J., Gomez F. (2020). A Regression Tree for Identifying Risk Factors for Fear of Falling: The International Mobility in Aging Study (IMIAS). J. Gerontol. Ser. A.

[44] Oviedo-Briones M., Laso Á.R., Carnicero J.A., Cesari M., Grodzicki T., Gryglewska B., Sinclair A., Landi F., Vellas B., Checa-López M. (2021). A Comparison of Frailty Assessment Instruments in Different Clinical and Social Care Settings: The Frailtools Project. J. Am. Med. Dir. Assoc..

[45] Gaboreau Y., Imbert P., Jacquet J.-P., Royer De Vericourt G., Couturier P., Gavazzi G. (2016). Barriers to and Promoters of Screening for Falls in Elderly Community-Dwelling Patients by General Practitioners: A Large Cross-Sectional Survey in Two Areas of France. Arch. Gerontol. Geriatr..

[46] Sum G., Nicholas S.O., Nai Z.L., Ding Y.Y., Tan W.S. (2022). Health Outcomes and Implementation Barriers and Facilitators of Comprehensive Geriatric Assessment in Community Settings: A Systematic Integrative Review [PROSPERO Registration No.: CRD42021229953]. BMC Geriatr..

[47] Yao S., Zheng P., Ji L., Ma Z., Wang L., Qiao L., Wan Y., Sun N., Luo Y., Yang J. (2020). The Effect of Comprehensive Assessment and Multi-Disciplinary Management for the Geriatric and Frail Patient: A Multi-Center, Randomized, Parallel Controlled Trial. Medicine.

[48] Belloni G., Büla C., Santos-Eggimann B., Henchoz Y., Fustinoni S., Seematter-Bagnoud L. (2021). Is Fear of Falling Associated With Incident Disability? A Prospective Analysis in Young-Old Community-Dwelling Adults. J. Am. Med. Dir. Assoc..

[49] Pereira C., Bravo J., Veiga G., Marmeleira J., Mendes F., Almeida G. (2020). Stepping-Forward Affordance Perception Test Cut-Offs: Red-Flags to Identify Community-Dwelling Older Adults at High Risk of Falling and of Recurrent Falling. PLoS ONE.

[50] Rathnayake N., Lekamwasam S. (2021). Prevalence and Factors Associated with Recurrent Falls among Middle-Aged Community-Dwelling Women. J. Frailty Sarcopenia Falls.

[51] Welch S.A., Ward R.E., Beauchamp M.K., Leveille S.G., Travison T., Bean J.F. (2021). The Short Physical Performance Battery (SPPB): A Quick and Useful Tool for Fall Risk Stratification Among Older Primary Care Patients. J. Am. Med. Dir. Assoc..

[52] Yoo I.-Y. (2011). Recurrent Falls Among Community-Dwelling Older Koreans: Prevalence and Multivariate Risk Factors. J. Gerontol. Nurs..

[53] Mazya A.L., Garvin P., Ekdahl A.W. (2019). Outpatient Comprehensive Geriatric Assessment: Effects on Frailty and Mortality in Old People with Multimorbidity and High Health Care Utilization. Aging Clin. Exp. Res..

[54] Romskaug R., Skovlund E., Straand J., Molden E., Kersten H., Pitkala K.H., Lundqvist C., Wyller T.B. (2020). Effect of Clinical Geriatric Assessments and Collaborative Medication Reviews by Geriatrician and Family Physician for Improving Health-Related Quality of Life in Home-Dwelling Older Patients Receiving Polypharmacy: A Cluster Randomized Clinical Trial. JAMA Intern. Med..

[55] Briggs R., McDonough A., Ellis G., Bennett K., O’Neill D., Robinson D. (2022). Comprehensive Geriatric Assessment for Community-Dwelling, High-Risk, Frail, Older People. Cochrane Database Syst. Rev..

